# Global Research Trends in Emerging Zoonosis Due to (the Filarial Nematode) *Dirofilaria repens* (1955–2025): A Bibliometric Analysis of a Climate-Driven Expansion

**DOI:** 10.3390/pathogens15040386

**Published:** 2026-04-03

**Authors:** Raúl Aguilar-Elena, Iván Rodríguez-Escolar, Manuel Collado-Cuadrado, Elena Infante González-Mohino, Alfonso Balmori-de la Puente, Alberto Gil-Abad, Rodrigo Morchón

**Affiliations:** 1 Research Group on Occupational Risk Prevention and Occupational Health and Safety (GPRL), Valencian International University, 46002 Valencia, Spain; 2 Zoonotic Diseases and One Health Group, Faculty of Pharmacy, Centre for Environmental Studies and Rural Dynamization (CEADIR), University of Salamanca, 37007 Salamanca, Spain; ivanrodriguez@usal.es (I.R.-E.); manuelcollado@usal.es (M.C.-C.); elena.igm4@usal.es (E.I.G.-M.); a.balmori@usal.es (A.B.-d.l.P.); albertogilabad@usal.es (A.G.-A.); 3 Biomedical Research Institute of Salamanca (IBSAL), University of Salamanca, 37007 Salamanca, Spain

**Keywords:** bibliometric analysis, climate change, *Dirofilaria repens*, emerging zoonosis, subcutaneous dirofilariosis, One Health

## Abstract

*Dirofilaria repens* is the leading cause of subcutaneous (dogs) and subcutaneous/ocular dirofilariosis (humans) in the Old World. Despite its rapid geographical spread, driven by climate change, the emergence of new invasive vectors (*Aedes albopictus*) and growing interest in its study due to the emergence of new cases in areas previously free of the parasite, amongst other factors, scientific research into this pathogen remains limited. This study provides the first longitudinal bibliometric analysis of global research on *D. repens* (1955–2025). Data from Web of Science and Scopus were processed using PRISMA and RAMIBS protocols, resulting in a normalized corpus of 624 documents analyzed via science mapping techniques. The field exhibits a sustained annual growth rate of 3.79%, transitioning into an exponential expansion phase in 2011. While Italy retains historical leadership, spatial analysis confirms a research displacement towards Central and Eastern Europe (Germany, Poland). Thematic evolution reveals a structural shift from isolated clinical case reports to a multidisciplinary ecosystem dominated by molecular epidemiology, vector competence, and surveillance. *Dirofilaria repens* has gone from being a minor and neglected issue to having a significant number of reports and studies subject to interest in addressing the disease that results from its infection in different hosts. However, the intellectual structure exposes an operational fragmentation between clinical medicine and medical entomology. Future research must overcome national silos and integrate reservoir management with vector control, transforming the current reactive approach into a predictive preventive system aligned with the One Health framework.

## 1. Introduction

*Dirofilaria repens* Railliet & Henry, 1911 (Rhabditida, Onchocercidae) is a filarial nematode that primarily affects domestic and wild carnivores, which act as reservoir hosts, and humans, who serve as accidental hosts. Adult worms of *D. repens* are typically located in the subcutaneous tissue and muscular fasciae of definitive hosts and frequently in ocular sites in humans. In animal reservoirs, adult worms release microfilariae into the bloodstream; when a vector (mosquitoes, Culicidae family) ingests blood, these microfilariae pass into the vector’s digestive tract, where they molt twice into infective third-stage larvae (L_3_). During a subsequent blood meal, these larvae penetrate the host through the bite wound and migrate to the subcutaneous tissue, where they evolve into the adult stage [1,2].

This parasite sits at a critical interface between animal, human, and environmental health, as its life cycle depends not only on the presence of canine reservoirs but also on ecological and climatic factors that favor interactions between competent vectors and susceptible populations in peri-urban and rural environments [2]. The vectors involved primarily belong to the genera *Aedes* Meigen, 1818, *Anopheles* Meigen, 1818 and *Culex* Linneo, 1758, whose vectorial competence is modulated by variations in temperature and humidity. In recent decades, climate change and habitat modification have facilitated the geographical expansion and seasonal activity period of these vectors, transforming what was historically considered a focal parasitosis into an expanding transboundary threat [3,4,5]. Specifically, the spread of invasive species such as *Aedes albopictus* Skuse, 1894 has created new transmission pathways in previously non-endemic temperate regions [6].

From a clinical perspective, the pathology presents divergent manifestations depending on the host. In dogs, the main reservoir, the infection is often asymptomatic or subclinical, although it can trigger dermatological conditions characterized by pruritus, erythema, subcutaneous nodules, and papular dermatitis, which frequently hinders early diagnosis and favors its silent dispersal. Conversely, in humans, it is the causative agent of subcutaneous/ocular dirofilariosis, typically manifesting as subcutaneous nodules or, with notable frequency, affecting ocular and conjunctival tissues, often requiring surgical intervention for parasite removal [7,8,9].

Epidemiologically, *D. repens* has established itself as an emerging zoonosis with a dynamic and rapidly expanding geographical distribution. Traditionally endemic in the Mediterranean basin, Eastern Europe, sub-Saharan Africa, and Asia, the parasite has shown a significant shift towards northern latitudes in recent years [5,8,9,10]. Currently, autochthonous cases are being reported in Central and Northern European countries (such as the Baltic states and Germany), where the infection was previously unknown [10,11]. This pattern of emergence turns subcutaneous dirofilariosis into a growing challenge for public health in Europe and Asia.

Despite its increasing zoonotic relevance and territorial expansion, *D. repens* has historically received less scientific attention compared to closely related parasites such as *D. immitis* Birago, 1626 (heartworm disease), often being categorized as a neglected parasite [12]. While systematic reviews on its biological prevalence exist, there is a notable gap in the literature regarding the analysis of scientific production itself [11,13,14,15]. To date, no global bibliometric studies have been conducted to examine the evolution of research on this pathogen, which prevents a clear understanding of how the scientific community has responded to its emergence, the identification of international collaboration networks, and the detection of persisting knowledge gaps.

Therefore, the objective of this study is to analyze the temporal and structural evolution of global scientific production on *D. repens* during the period 1955–2025 using advanced bibliometric indicators and science mapping techniques. The aim is to characterize research trends and visualize the conceptual structure of the field, allowing for an evaluation of the needs regarding this emerging zoonosis and providing a solid documentary basis to guide future control strategies under a One Health perspective.

## 2. Materials and Methods

### 2.1. Study Design and Methodological Framework

A descriptive and retrospective study was conducted through a bibliometric analysis of the global scientific production related to *D. repens*. To guarantee reproducibility and scientific rigor, the research was structured under the Reporting and Measurement of Items for Bibliometric or Scientometric Studies in Health Sciences (RAMIBS) methodology [16], ensuring data integrity and a synthetic analysis of the evolutionary trends in the field. Detailed compliance with each integrity item required by this protocol is provided in Supplementary Material Table S1 (RAMIBS Checklist). The workflow was designed to ensure transparency and reproducibility and was reported following the principles of the Preferred Reporting Items for Systematic Reviews and Meta-Analyses (PRISMA) statement [17], specifically adapted for bibliometric studies.

### 2.2. Search Strategy and Data Sources

Information retrieval was carried out in January 2026 using the Web of Science (WoS) (Core Collection) and Scopus databases. A search strategy was designed to maximize the recall of scientific literature regarding the parasite. The search equations used were: Scopus: TITLE-ABS-KEY(“*Dirofilaria repens*” OR “subcutaneous dirofilariasis”) and Web of Science: TS=(“*Dirofilaria repens*” OR “subcutaneous dirofilariasis”). Although the primary search focused on the specific taxon, the clinical term “subcutaneous dirofilariasis” was included to capture human case reports where the etiological agent is identified in the body of the text but not necessarily in the title by its scientific name. No language or document type filters were applied to avoid selection bias, allowing for a global view of the field’s evolution from 1955 to 2025.

### 2.3. Study Selection and Data Normalization (Inclusion Criteria)

Records were included if they met the following criteria: original articles, reviews, or scientific notes focused on *D. repens*, published between 1955 and 2025, without language restriction. Studies focused exclusively on *D. immitis* were excluded; however, comparative studies and coinfection reports were retained to preserve the ecological and diagnostic context of the species. Following inclusion, metadata normalization was performed to standardize terminological variants in keywords (e.g., “Dirofilariosis” vs. “Dirofilariasis”) and to unify institutional affiliations, ensuring consistency across the analytical corpus.

### 2.4. Bibliometric Analysis and Visualization

Quantitative analysis was performed using the biblioshiny interface, structuring the evaluation into three hierarchical levels: (1) scientific activity indicators were calculated, such as the annual growth rate, author productivity (Lotka’s Law), and source impact (Bradford’s Law), and the geographic distribution was analyzed to identify the shift in research towards Central and Northern Europe; (2) co-authorship networks between countries and institutions were generated to evaluate the degree of cooperation under the One Health approach, analyzing the intersection between human and veterinary medicine; and (3) thematic maps based on density and centrality were employed to categorize topics into motor, basic, or emerging themes. Finally, a thematic evolution analysis was conducted using time slices to visualize the transition from initial clinical case reports to current frontiers in molecular diagnosis and environmental epidemiology. Network visualizations were optimized using the Fruchterman-Reingold algorithm to ensure the readability of the identified clusters and thematic interconnections.

### 2.5. Study Selection Flowchart

**Figure 1:**
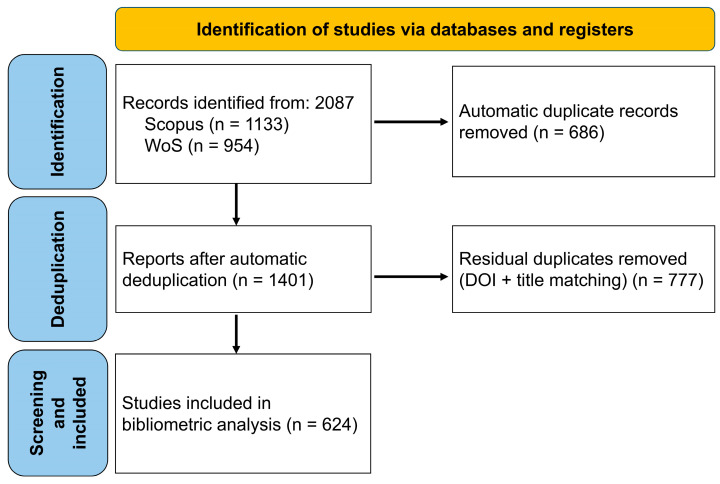
PRISMA flow diagram of record identification, screening, and inclusion for the bibliometric corpus on *Dirofilaria repens* (Scopus and Web of Science Core Collection).

The literature identification and selection process was reported following the PRISMA 2020 statement adapted for bibliometric studies, ensuring transparency in the formation of the study corpus (Figure 1). In the identification phase, 2087 raw records were retrieved from Scopus (n = 1133) and Web of Science (n = 954). During the first deduplication step, 686 duplicate records were automatically eliminated using the mergeDbSources function of the bibliometrix package, yielding 1401 records. A second hard deduplication step based on DOI cross-matching and normalized title comparison removed a further 777 residual duplicates, resulting in 624 unique records assessed for eligibility. The conceptual filter described in Section 2.3 was then applied; no additional records were excluded at this stage (n = 0), as the search strategy terms (“*Dirofilaria repens*”/“subcutaneous dirofilariasis”) already ensured sufficient taxonomic specificity. The final dataset consisted of 624 documents (1955–2025), which was used for all subsequent performance analyses, scientific mapping, and thematic evolution. The entire process was conducted following the RAMIBS recommendations to ensure transparency and reproducibility.

## 3. Results

### 3.1. Evolution of Scientific Production and Source Analysis

**Figure 2:**
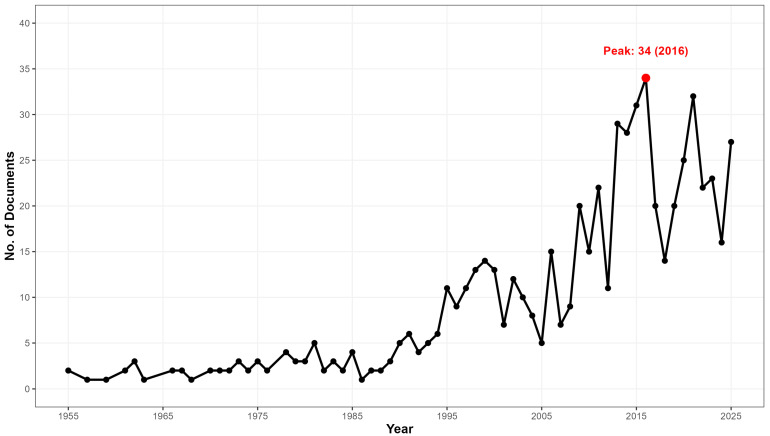
Annual scientific production on *Dirofilaria repens* (1955–2025).

Research activity on *D. repens* shows an upward trajectory from the first identified record in 1955 to 2025. The analyzed corpus of 624 documents reflects an annual growth rate of 3.79% (Table 1), evidencing sustained scientific interest. This growth allows for the identification of three evolutionary phases (Figure 2, Table S1). The first period, or latency phase (1955–1990), was characterized by marginal production (average < 3 docs/year), with only 5 documents recorded in 1990. This was followed by a growth and consolidation phase (1991–2010), a period of clinical knowledge expansion that closed in 2010 with 15 annual publications. Finally, the field entered an Exponential Expansion Phase (2011–2025), which concentrates the highest volume of production, reaching its peak in 2016 with 34 documents. This increase coincides temporally with the alerts regarding the parasite’s expansion towards northern latitudes and the rise in human cases reported in Eastern Europe.

Regarding the scientific structure of impact, the study reveals a high level of professionalization in the field, with an average of 5.29 co-authors per document and 16.19% international collaboration. Global impact is reflected in an average of 13.43 citations per document, highlighting the works of Pampiglione [1] (252 citations) and Capelli [10] (216 citations) as pillars in the epidemiology and diagnosis of the disease. In terms of sources, documents are distributed across 277 journals. However, the application of Bradford’s Law identifies a highly specialized core (Zone 1) composed of 18 journals. This core is led by *Parasitology Research* (31 articles), *Veterinary Parasitology* (28), and *Parasites & Vectors* (21). The presence of journals such as *Emerging Infectious Diseases* and the *American Journal of Tropical Medicine and Hygiene* in this core reinforces the zoonotic and public health character of current research.

### 3.2. Geographical Distribution and International Collaboration Networks

The analysis of production by country reveals a significant concentration in historically endemic regions, with a clear transition towards a global cooperation model (Table 2). Italy stands as the undisputed leader in this field and the highest recorded scientific impact, accumulating 2344 citations with an average of 26.6 citations per article (Table 2). Following Italy’s leadership, India has consolidated itself as the main research focus outside Europe. In the European context, Germany and Poland reflect the importance of Central and Eastern European countries in studying the parasite’s expansion, while France and Spain complete the block of Mediterranean basin countries with high research activity.

In terms of international collaboration, the study shows a rate of 16.19%. Although there is a considerable volume of domestic publications (SCP—Single Country Publications), countries such as Spain (MCP Ratio: 0.44) and Germany (MCP Ratio: 0.34) show a trend well above the average in creating transnational networks. This cooperation is articulated mainly through European clusters connecting institutions in Italy, Germany, and the Balkans, suggesting a coordinated scientific response to the expansion of dirofilariosis towards the north of the continent. The citation analysis by country reinforces this structure: while Italy dominates in volume, countries such as Switzerland (37.4 citations/article) and Austria (20.2 citations/article) present a very high relative impact, indicating that their contributions, although fewer in number, are fundamental to the international scientific community (Table 2).

### 3.3. Thematic Analysis and Research Evolution

**Figure 3:**
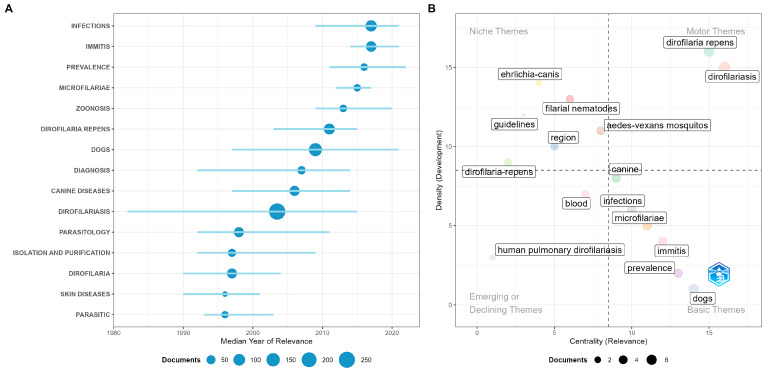
Conceptual structure and thematic evolution of *Dirofilaria repens* research. (**A**) Thematic evolution trends. The bubble size represents the document frequency, while the horizontal lines indicate the temporal span of prominence for each term. (**B**) Strategic thematic map.

The conceptual evolution of *D. repens* reflects a transition from a discipline based on clinical observation to an integrated and molecular science (Figure 3). Historically, the field was grounded on general terms such as “Dirofilariasis” (which groups the older “Filariasis”), “Parasitology,” and “Isolation and Purification,” with a markedly dermatological and descriptive focus centered on “Skin Diseases” during the 1990s. However, the thematic map analysis reveals that the dynamic nucleus or “motor” of current research has matured significantly, positioning the dog (“Dogs”) and canine disease (“Canine Diseases”) as the pillars of greatest development and strategic centrality. This shift towards veterinary medicine as the basis of public health consolidated between 2005 and 2015, a period in which the term “Diagnosis” gained volume, evidencing an effort to systematize the detection of the parasite beyond isolated surgical case reports.

At the frontier of current knowledge, the field shows a strong trend towards global epidemiological surveillance, where “Zoonosis,” “Prevalence,” and “Infections” appear as the terms with the highest growth in the last decade. It is particularly relevant that, currently, research on *D. repens* is not conducted in isolation but under a comparative lens with cardiopulmonary dirofilariosis to understand joint geographical expansion patterns. Meanwhile, the emergence of specialized niches focused on specific vectors such as *Culex pipiens* and coinfections with pathogens like *Ehrlichia canis* Donatien & Lestoquard, 1935 suggests increasing technical sophistication. Finally, the positioning of techniques such as “Real-time PCR” in the transition quadrant towards motor themes confirms that the future of the discipline lies in molecular standardization, definitively shifting interest from human clinical case reporting—now in relative decline—towards integral epidemiological control under the One Health approach.

### 3.4. Intellectual Organization and Conceptual Structure of the Field

**Figure 4:**
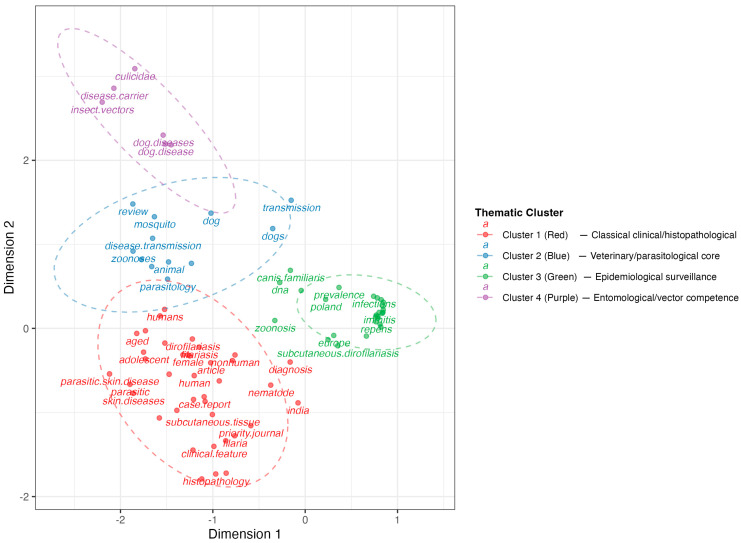
Intellectual organization and conceptual clustering of *Dirofilaria repens* research based on multiple correspondence analysis. The conceptual structure map presents a two-dimensional representation where the spatial positioning of keywords reflects their thematic proximity and co-occurrence pattern.

The conceptual structure of *D. repens* research, as revealed by Multiple Correspondence Analysis, identifies four distinct thematic domains (Figure 4). Cluster 1 (red) represents the historical foundation of the field, grouping clinical and histopathological literature centered on case reports, skin diseases, and subcutaneous manifestations in human patients. Cluster 2 (blue) constitutes the veterinary and parasitological core, where the dog as primary reservoir converges with concepts of zoonotic transmission, animal parasitology, and vector-host dynamics. Cluster 3 (green) reflects the epidemiological surveillance frontier, integrating geographic expansion towards Central Europe (Poland), prevalence data, molecular identification (DNA), and the co-circulation of *D. repens* and *D. immitis* in areas of sympatry. Cluster 4 (purple) stands apart, dedicated exclusively to vector biology and entomological competence (culicidae, insect vectors, disease carriers).

**Figure 5:**
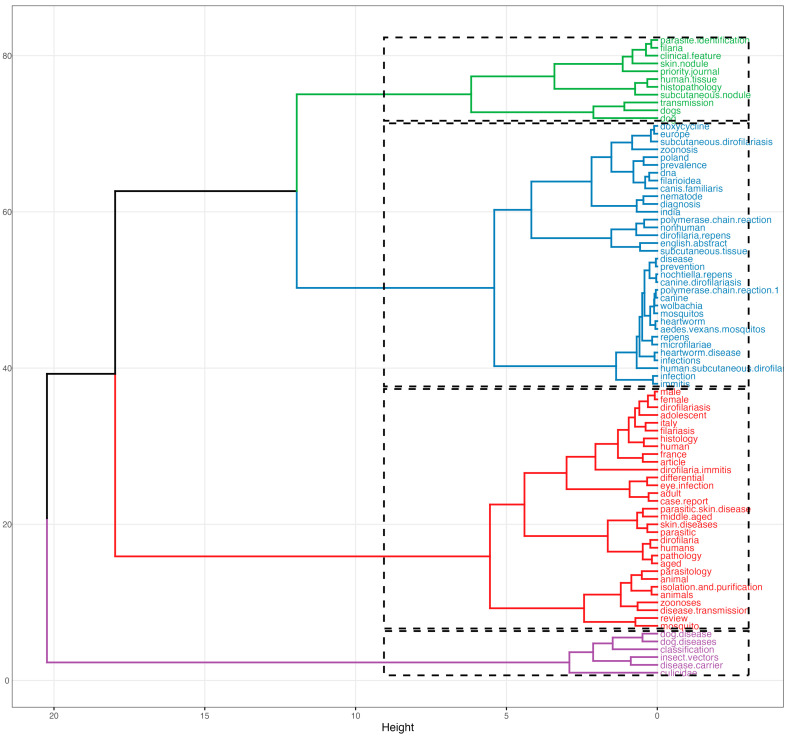
Topic dendrogram of *Dirofilaria repens* research themes based on hierarchical clustering.

The hierarchical dendrogram (Figure 5) supports a four-domain organization of the field and reveals the genealogical relationships between these thematic areas. The most significant finding is the early separation of the cluster 4 (vector biology: culicidae, insect vectors, disease carriers) from the 1–3 clusters, which share a common root. This separation pattern reflects a structural disconnect between entomological research (purple cluster) and the clinical-epidemiological mainstream (green, blue and red cluster) that has characterized the field throughout its history and that continues to limit the full operationalization of a One Health approach to *D. repens* surveillance and control.

## 4. Discussion

The results of this bibliometric analysis confirm that research on *D. repens* has undergone a paradigm shift, transitioning from being regarded as a purely individual and localized issue. The exponential acceleration of scientific production observed since 2011 reflects an upward trend in the number of investigations and interest in the disease. While Italy maintains its historical hegemony as the epicenter of knowledge, the dynamism observed in the Poland-Germany-Russia axis has significantly increased the number of studies on D. repens [2,5,8,9,10,18,19].

Studies conducted over the last two decades show that both the study of the disease and the emergence of new cases or imported infected hosts in a given country may be directly linked to the increased interest in researching the disease, driven by the emergence of new vectors, the rise in travel with pets, and rising temperatures, amongst other factors [20,21]. In Central European countries, where the number of reported cases has risen significantly, cases historically classified as imported are now increasingly interpreted as evidence of emerging local transmission, a phenomenon linked to the adaptation of competent vectors to urban microclimates and the urban heat island effect [6,22,23].

Although the One Health approach is postulated as the theoretical pillar of this field, our analysis reveals significant operational fragmentation. While the diagnostic Technification and the human clinic demonstrate notable technological maturity—integrating everything from advanced ophthalmological surgery to genomic sequencing—the hierarchical dendrogram exposes an early divergence between these clinical fields and medical entomology. This disconnection suggests that a large part of the scientific community addresses the human symptom (subcutaneous or ocular nodule) in isolation, without real integration with canine reservoir management and vector control [23]. Furthermore, there is a low level of international collaboration within the scientific community, which could also be linked to cross-border spread from a One Health perspective [12].

A crucial finding of this study is the position of *D. immitis* as a transversal theme within the conceptual map of *D. repens*. Historically, *D. repens* has remained in the shadow of cardiopulmonary dirofilariosis due to the latter’s greater clinical severity in dogs [11,20]. However, this carries a risk of diagnostic bias in areas of sympatry. The advent of real-time PCR has enabled differentiation between the two species in diagnosis, leading to the identification of many more cases of *D. repens* infection, in contrast to conventional serological tests which could not distinguish between the two parasites due to cross-reactivity [24]. Whilst *D. immitis* causes vascular and pulmonary lesions in canids and felids, and occasionally pulmonary lesions in humans, *D. repens* presents with greater dermatological morbidity in animals and, furthermore, ophthalmological morbidity in humans, which frequently requires surgical intervention [2,4,11].

Following studies on the prevalence of the disease, in terms of both veterinary and human diagnosis, a new approach to its study has begun to emerge, focusing on research using predictive models, which could bring about a transformation in the field of research. In this regard, it may become necessary to prioritize the integration of habitat suitability models for its vectors with data on the presence of infectious vectors and their density to anticipate new outbreaks, thereby transforming the current, predominantly reactive approach [5].

Finally, certain limitations of the study must be acknowledged. The predominance of English literature could undervalue scientific production in vernacular languages from regions with a high endemic burden. Likewise, citation inertia may favor classic works from the consolidation phase, delaying the reflection of the disruptive impact of more recent research on ecological modeling.

## 5. Conclusions

This bibliometric analysis demonstrates that *D. repens* infection has changed its historical status from being a neglected or under-analyzed parasitic disease to becoming a disease of interest, with an increase in the number of reports and studies in recent years. The documentary data reveal a shift in publication patterns and an increase in scientific output, which may be due to growing interest in studying the disease driven by various factors. While the discipline has reached a level of technological maturity that allows for precise molecular diagnosis and accurate differentiation from other filariae in areas of sympatry, a notable operational fragmentation persists, limiting the real efficacy of the One Health approach. Therefore, it is important to continue promoting transnational cooperation networks that integrate entomological surveillance and canine control of reservoirs, transforming the scientific response into a preventive system capable of anticipating the spread of this zoonotic pathogen.

## Figures and Tables

**Table 1:** Main information and bibliometric indicators of the scientific production on *Dirofilaria repens* (1955–2025). Data retrieved from Web of Science and Scopus (January 2026).

Indicator	Value
*Main information*	
Timespan	1955:2025
Sources (journals, books, etc)	277
Documents	624
Annual growth rate %	3.79%
Document average age	17.9
Average citations per doc	13.43
*Document contents*	
Keywords plus	1052
Author’s keywords	671
*Authors and collaboration*	
Total authors	2474
Authors of single-authored docs	34
Co-authors per doc	5.29
International co-authorships %	16.19%

**Table 2:** Top 10 most productive countries in *Dirofilaria repens* research and international collaboration patterns (1955–2025). SCP: Single Country Publications (publications where all authors belong to the same country); MCP: Multiple Country Publications (publications with authors from at least two different countries); MCP Ratio: the proportion of international collaboration relative to the country’s total production. Data sorted by total number of documents.

Country	Total Documents	SCP	MCP	MCP Ratio
Italy	88	68	20	22.7%
India	49	47	2	4.1%
Germany	38	25	13	34.2%
Poland	38	31	7	18.4%
France	32	30	2	6.3%
USA	20	15	5	25.0%
Spain	18	10	8	44.4%
Austria	12	11	1	8.3%
Croatia	12	11	1	8.3%
Hungary	12	10	2	16.7%

## Data Availability

The original contributions presented in this study are included in the article. Further inquiries can be directed to the corresponding authors.

## Supplementary Materials

The following supporting information can be downloaded at: https://www.mdpi.com/article/10.3390/pathogens15040386/s1, Table S1: Compliance with the Reporting and Measurement of Items for Bibliometric or Scientometric Studies in Health Sciences (RAMIBS) recommendations in the present bibliometric study on Dirofilaria repens global research trends (1955–2025).
