# Supplementary material for: Global Research Trends in Emerging Zoonosis Due to (the Filarial Nematode) Dirofilaria repens (1955–2025): A Bibliometric Analysis of a Climate-Driven Expansion
Source: Pathogens. 2026 Apr 3;15(4):386. doi: 10.3390/pathogens15040386 (PMC13118602; doi:10.3390/pathogens15040386)
Supplement: Supplementary file 1 [file pathogens-15-00386-s001.zip › pathogens-4180818-supplementary.pdf]

Table S1. Compliance with the Reporting and Measurement of Items for Bibliometric or Scientometric Studies in Health Sciences (RAMIBS) recommendations in the present bibliometric study on *Dirofilaria repens* global research trends (1955–2025).

RAMIBS item	Recommendation	Compliance	Section / Description
1. Title and abstract	Explanatory title reflecting the bibliometric approach; structured abstract	Yes	**Title:** ' Global Research Trends on the Emerging Zoonosis Dirofilaria repens (1955–2025): A Bibliometric Analysis of a Climate-Driven Expansion, explicitly identifying the subject and approach. Structured abstract includes objectives, databases (WoS/Scopus), period, and key findings.
2. Introduction	Antecedents, relevance, research question, justification, and objectives	Yes	The introduction contextualizes *D. repens* as an emerging zoonosis, identifies the lack of previous bibliometric evidence (PICO gap), and explicitly defines research justification and objectives.
3. Databases	Justification of database selection; inclusion/exclusion criteria; time period; deduplication	Yes	Web of Science Core Collection and Scopus were selected for their high-quality indexing. Inclusion criteria, time period (1955–2025), and deduplication procedures (DOI + Title normalization) are detailed in the Methods.
4. Search strategy	Detailed construction of search strategy, Boolean operators, and field selection	Yes	The search strategy was built using terms 'Dirofilaria repens' and 'subcutaneous dirofilariasis', applied across databases using Boolean operators in Title, Abstract, and Keyword fields.
5. Search filters	Exact search date, document types, language restrictions	Yes	The specific search date (January 2026), document types (articles/reviews), and absence of language restrictions are detailed to ensure reproducibility.
6. Bibliometric indicators	Use of core bibliometric dimensions (production, impact, collaboration, thematic)	Yes	Indicators of production (annual growth), impact (citations), collaboration (MCP/SCP ratios), and thematic structure (Thematic Map/MCA) were analyzed using the *bibliometrix* package.
7. Unit of analysis	Macro (countries), meso (institutions/topics), micro (authors)	Yes	Analysis was performed at macro (global trends/countries), meso (journals/thematic clusters), and micro (most relevant authors) levels.

RAMIBS item	Recommendation	Compliance	Section / Description
8. Data extraction and processing	Deduplication, normalization, analytical techniques, justification	Yes	Records were merged and deduplicated via DOI matching and title normalization. Data cleaning was performed for keywords and country names (e.g., UK/USA normalization) before analysis in R.
9. Presentation of data	Software versions, visualization parameters, normalization procedures	Yes	Data visualization was performed via *bibliometrix* (R), using standardized parameters (Association Strength) for networks and 300 DPI high-resolution TIFF format for figures.
10. Results	Transparent presentation, interpretation, excluded records	Yes	Results are presented through 5 core figures with corresponding interpretation. A PRISMA-style accounting of excluded records (conceptual filter for *D. immitis* vs *D. repens*) is provided.
11. Discussion	Interpretation, comparison with other bibliometric studies, limitations	Yes	Results are discussed in relation to the 'thermal footprint' of the parasite and the One Health framework, highlighting geographic expansion and acknowledging limitations (database bias).
12. Conclusion	Summary of main findings	Yes	The conclusion synthesizes the identified patterns, focusing on the bimodal evolution of research and the need for cross-border entomological-clinical collaboration.
